# Are We Ready For “Triplet” Therapy in Higher-Risk MDS?

**DOI:** 10.46989/001c.88301

**Published:** 2023-11-03

**Authors:** Andrew M. Brunner, Uwe Platzbecker, Amy E. DeZern, Amer M. Zeidan

**Affiliations:** 1 Leukemia Program, Massachusetts General Hospital Cancer Center, Harvard Medical School, Boston, MA, USA; 2 Leipzig University Hospital, Leipzig, Germany; 3 Sidney Kimmel Comprehensive Cancer Centre at John Hopkins, Baltimore, MD, USA; 4 Section of Hematology, Department of Internal Medicine, Yale University School of Medicine and Yale Cancer Center, New Haven, CT, USA.

**Keywords:** myelodysplastic syndromes, azacitidine, decitabine, allogeneic transplant, combination chemotherapy

## Abstract

Higher-risk Myelodysplastic Syndromes/Neoplasms (MDS) represent an ongoing therapeutic challenge, with few effective therapies, many of which may have limited use in this older patient population often with considerations around comorbidities. Outside of transplant, azacitidine and decitabine remain the only disease-modifying therapies, and are palliative in nature. Recent interest has grown in extending combination chemotherapies used to treat acute myeloid leukemia (AML) to patients with MDS, including novel combination chemotherapy “doublets” and “triplets.” In this review, we discuss considerations around combination chemotherapy in MDS, specifically as relates to study design, appropriate endpoints, supportive considerations, and how to integrate these into the current treatment paradigm. New therapies in MDS are desperately needed but also require considerations particular to this unique patient population.

## Introduction

The approach to the treatment of patients with myelodysplastic syndromes/neoplasms (MDS) generally depends on the expected course of disease following diagnosis, or as disease evolves during treatment.^1–3^ Patients whose MDS has features suggesting it is likely to be a chronic co-morbid illness are generally treated with supportive therapies directed at symptomatic cytopenias. MDS with these features is generally classified as “lower-risk” (LR-MDS), indicating a lower overall risk of death from MDS or progression to acute myeloid leukemia (AML) in the immediate years after diagnosis.^4^ In contrast, MDS that has worse features such as deep cytopenias, increasing blasts, and poor risk cytogenetic or molecular alterations, may be classified as higher-risk MDS (HR-MDS), and is associated with poor overall survival and increased rates of progression to AML within months to a few years after initial diagnosis.^5^

For patients with HR-MDS, the goal of therapy focuses instead on improving both the quantity and quality of life after diagnosis.^6^ A number of risk scores are employed to characterize MDS disease risk, which continue to evolve as we gain better understanding of the pathobiology of MDS and its relation to other myeloid neoplasms. Nonetheless, identifying these patients with higher-risk disease features is important, as they should be considered for therapies that alter the natural course of disease, which include DNA methyltransferase inhibitors (DNMTIs) such as azacitidine or decitabine,^7,8^ or allogeneic hematopoietic cell transplantation, the only potentially curative therapy for his malignancy.^9,10^

While DNMTIs may prolong overall survival and improve the quality of life of patients with HR-MDS,^7,11,12^ their impacts are palliative in nature, a minority of patients achieves complete responses, and essentially all patients will eventually relapse or progress with their disease, after which survival is particularly poor.^13–15^ Real-life studies have generally reported a median overall survival of only 11-19 months with DNMTI therapy for patients with HR-MDS.^16–18^ Furthermore, there is no appreciable tail at end of survival curve following DNMTi therapy without transplantation, and within five years nearly all patients will have died,^19^ prompting ongoing urgent need for new therapeutics in this space. Once such focus has been on the use of combination therapies utilizing a DNMTI “backbone” to improve upon DNMTI monotherapy, with the goal of improving the rate of responses, quality/depth or duration of responses, overall survival, or some combination of the above (Table 1). While several randomized phase III studies are underway exploring “doublets” which may improve upon DNMTI monotherapy (NCT04266301, NCT04401748, NCT04313881),^20–22^ already there is interest in further combinations including “triplet” therapy in the treatment of MDS.

**Table 1.** Trials Evaluating DNMTI Combinations in MDS. Shown are studies on clinicaltrials.gov which are recruiting, not yet recruiting, active not recruiting, or enrolling by invitation.

**NCT Number**	**Title**	**Interventions**
**NCT04878432**	STIMULUS MDS-US : Sabatolimab Added to HMA in Higher Risk MDS	Drug: MBG453|Drug: Azacitidine|Drug: Decitabine|Drug: INQOVI (oral decitabine)
**NCT03092674**	Azacitidine With or Without Nivolumab or Midostaurin, or Decitabine and Cytarabine Alone in Treating Older Patients With Newly Diagnosed Acute Myeloid Leukemia or High-Risk Myelodysplastic Syndrome	Drug: Azacitidine|Drug: Cytarabine|Drug: Decitabine|Other: Laboratory Biomarker Analysis|Drug: Midostaurin|Biological: Nivolumab
**NCT04730258**	A Study of CFI-400945 With or Without Azacitidine or Decitabine in Patients With AML, MDS or CMML	Drug: CFI-400945|Drug: Azacitidine|Drug: Decitabine
**NCT03066648**	Study of PDR001 and/or MBG453 in Combination With Decitabine in Patients With AML or High Risk MDS	Drug: Decitabine|Drug: PDR001|Drug: MBG453|Drug: Azacitidine
**NCT04146038**	Salsalate, Venetoclax, and Decitabine or Azacitidine for the Treatment of Acute Myeloid Leukemia or Advanced Myelodysplasia/Myeloproliferative Disease	Drug: Azacitidine|Drug: Decitabine|Drug: Salsalate|Drug: Venetoclax
**NCT05564650**	Evaluating Navitoclax After Failure of Standard Treatments of Azacitidine or Decitabine and Venetoclax in Patients With Aggressive Myelodysplastic Syndrome	Biological: Navitoclax|Drug: Venetoclax|Drug: Decitabine|Procedure: Bone Marrow Biopsy|Procedure: Biospecimen Collection|Other: Laboratory Biomarker Analysis|Other: Quality-of-Life Assessment
**NCT05426798**	Clinical Study of TQB2618 Injection in Combination With Demethylation Drugs in Patients With Recurrent/Refractory Acute Myeloid Leukemia, Myelodysplastic Syndromes	Drug: TQB2618 injection azacitidine, AZA decitabine, DAC
**NCT04358393**	A Study of APG-115 Alone or Combined With Azacitidine in Patients With AML, CMML, or MDS	Drug: APG-115|Drug: 5-azacitidine
**NCT05766514**	Phase II Prospective Randomized Control Trial of Cladribine and Low-Dose Cytarabine (LoDAC) Alternating With Decitabine vs. Hypomethylating Agents (HMA) Plus Venetoclax as Frontline Therapy for AML or High-Grade MDS in Patients Unfit for Intensive Induction	Drug: Cladribine|Drug: Cytarabine|Drug: Decitabine|Drug: azacitadine or decitabine|Drug: Venetoclax
**NCT05184842**	Metabolically Optimized, Non-cytotoxic Low Dose Weekly Decitabine/Venetoclax in MDS and AML	Drug: Venetoclax|Drug: Decitabine
**NCT03946670**	A Study of MBG453 in Combination With Hypomethylating Agents in Subjects With IPSS-R Intermediate, High or Very High Risk Myelodysplastic Syndrome (MDS).	Drug: MBG453|Drug: Placebo|Drug: Hypomethylating agents
**NCT03164057**	A Trial of Epigenetic Priming in Patients With Newly Diagnosed Acute Myeloid Leukemia	Drug: Azacitidine|Drug: Decitabine|Drug: Cytarabine|Drug: Daunorubicin|Drug: Etoposide|Combination Product: ITMHA|Drug: Idarubicin|Drug: Fludarabine|Drug: Mitoxantrone|Drug: Erwinia asparaginase|Drug: Sorafenib|Drug: G-CSF|Drug: Dexrazoxane|Biological: Stem Cell Transplant|Drug: Asparaginase Erwinia Chrysanthemi, Recombinant-Rywn
**NCT02890329**	Ipilimumab and Decitabine in Treating Patients With Relapsed or Refractory Myelodysplastic Syndrome or Acute Myeloid Leukemia	Drug: Decitabine|Biological: Ipilimumab
**NCT03404193**	Venetoclax and Decitabine in Treating Participants With Relapsed/Refractory Acute Myeloid Leukemia or Relapsed High-Risk Myelodysplastic Syndrome	Drug: Decitabine|Other: Laboratory Biomarker Analysis|Drug: Venetoclax
**NCT03969446**	Pembrolizumab and Decitabine With or Without Venetoclax in Treating Patients With Acute Myeloid Leukemia or Myelodysplastic Syndrome That Is Newly-Diagnosed, Recurrent, or Refractory	Drug: Decitabine|Biological: Pembrolizumab|Drug: Venetoclax
**NCT03661307**	Quizartinib, Decitabine, and Venetoclax in Treating Participants With Untreated or Relapsed Acute Myeloid Leukemia or High Risk Myelodysplastic Syndrome	Drug: Decitabine|Drug: Quizartinib|Drug: Venetoclax
**NCT04282187**	Decitabine With Ruxolitinib or Fedratinib for the Treatment of Accelerated/Blast Phase Myeloproliferative Neoplasms	Drug: Decitabine|Drug: Ruxolitinib|Drug: Fedratinib|Other: Questionnaire Administration
**NCT02085408**	Clofarabine or Daunorubicin Hydrochloride and Cytarabine Followed By Decitabine or Observation in Treating Older Patients With Newly Diagnosed Acute Myeloid Leukemia	Drug: Daunorubicin|Drug: Cytarabine|Drug: Clofarabine|Drug: Decitabine|Other: Observation|Procedure: Allogeneic hematopoietic stem cell transplantation

In this manuscript we evaluate considerations around MDS therapies as novel combinations, including triplet combination therapies, are increasingly evaluated. This includes the need to better characterize what “higher-risk” MDS entails in a modern era, what endpoints are most relevant for trials in MDS, the impact of trial designs, the expected divergence between the highly selected patients who could potentially tolerate combination therapies versus the typically older and more frail patients seen in clinics, and ways in which combination therapies may become new standards of care.

## What constitutes higher-risk MDS in the current era?

An important consideration for studies evaluating higher risk MDS, is to consider how the patients included on such studies may impact the expected outcomes from the intervention. Such differences may be balanced in the randomized setting, but single arm studies may be more prone to influences by the patient composition.^23^ Risk assessment has evolved significantly over the last 25 years, and these changes should be considered both when selecting patients for clinical trial enrollment,^24–26^ as well as when determining appropriate response outcomes. At the same time, there have been significant changes in the classification of new MDS diagnoses, now incorporating more extensive molecular and cytogenetic information along with histomorphologic evaluation into the subclassification of this malignancy.^27,28^ Because clinical trial inclusion criteria typically involve both specific MDS subtypes as well as specific risk groups in their eligibility, changes in either the diagnostic or prognostic criteria used in MDS studies may impact expected outcomes.^29^

Risk stratification with the molecular international prognosis scoring system (IPSS-M) incorporates molecular data using the presence, absence, and other features of somatic mutations identified at MDS diagnosis.^26^ It builds upon prior work to develop the original IPSS^24^ as well as the revised IPSS (IPSS-R)^25^ and indeed has an improved predictive capacity for overall survival and the cumulative incidence of progression to AML. However, there are specific criteria that should be considered particularly in early-stage combination therapy development. Pivotal trials leading to the approval of azacitidine and decitabine enrolled patients typically with higher-risk disease as defined by the IPSS, which gives less weight to the depth of specific cytopenias compared to the IPSS-R, and also had fewer cytogenetic risk categories.^30^ The IPSS was also developed at a time when the classification of MDS included patients with 20-30% bone marrow blasts,^31^ a group who would now be classified as AML. The IPSS-M continues to utilize the cytogenetic risk groups identified in the larger sample size of the IPSS-R score, but the addition of molecular alterations resulted in the risk re-stratification of 46% of patients.^26^

Further complicating risk stratification and classification in MDS is the increasing overlap between MDS and AML, particularly as relates to blast counts and specific genetic alterations that define AML or MDS. In the most recent updates, patients with 10-19% blasts are considered to have MDS/AML according to the ICC,^27^ which may match biological understanding of these malignancies, but does create some challenges for trial development. Similarly, patients with lower bone marrow blast counts (<20%) typically considered to have MDS may now be re-classified as AML if their disease harbors typical AML mutations such as in *NPM1*.^27,28^ It is important to consider how these may impact expected trial results; for instance, increasing the proportion of patients on a trial whose disease is considered high risk due to molecular profile, but without increased blasts, may diminish the number of patients who are eligible for a complete remission or other marrow responses, which still largely depend on blast reduction. At the same time, a cohort with a higher risk of leukemia progression or lower overall survival may be better suited to assess one of these as the primary outcome. In addition, if studies are established based on IPSS-M results, there are practical considerations around obtaining these results that may impact the translation of study findings into clinical practice. This includes the selection of an appropriate molecular panel which captures the genes to calculate the IPSS-M, the turnaround time for the assay, and additional resources needed to conduct such testing – which may not be readily available depending on local resources.^32^

## Defining the current standard of care in higher-risk MDS – is there a doublet to build on?

When considering triplet regimens in HR-MDS, it is important to consider whether there is an existing doublet which would serve as the backbone. To date, no doublet has shown superiority to azacitidine monotherapy in prolonging overall survival for the treatment of HR-MDS patients in a randomized trial.^33–35^ In the AZA-001 trial, azacitidine showed benefit compared to conventional care, which included a proportion patients treated with intensive chemotherapy.^7^ A total of 42 patients were pre-selected for intensive chemotherapy, and this subgroup, while underpowered, had overlapping survival confidence intervals with azacitidine, implying that treatment intensification may make sense in a subset of patients. Arguably, most doublets with a DNMTI backbone result in “intensified” treatment often mirroring strategies employed in AML. In considering a triplet regimen, it is thus important to consider overlapping toxicities particularly as relates to the profound cytopenias often seen in HR-MDS.

There are currently four late phase randomized trials evaluating azacitidine as monotherapy or in combination with another agent, including the BCL2 inhibitor venetoclax (NCT04401748), the CD47 inhibitor magrolimab (NCT04313881), the TIM3 inhibitor sabatolimab (NCT04266301), and the retinoid tamibarotene (NCT04797780). Each of these studies is underway with results anticipated in the coming years, including evaluation of differences in response rates, as well as overall and leukemia free survival. Should any be approved, this could clarify future steps in triplet development, although a number of questions will remain including sequencing of therapies, treatment discontinuation considerations, and timing to enhance synergistic benefit. Recent randomized studies in MDS underscore some of the uncertainty in this calculus; for instance, had triplet regimens been tested based on early data with eprenetapot (APR-246) or pevonedistat,^36,37^ it would be difficult to interpret subsequent findings, particularly if the studies did not show any significant benefit.

In lieu of a new approval, a question remains as to which backbone doublet should be used to evaluate any “triplet” regimens at this time. Arguably, the one area where this may be possible, particularly given the recent changes in MDS and AML classification discussed above, would be as relates to the doublet of azacitidine and venetoclax.^22,38^ Azaciditine combined with venetoclax is approved for the treatment of patients with AML who are not candidates for intensive induction chemotherapy.^39^ It has been studied both in the frontline setting in MDS, where early phase studies suggest relatively higher response rates compared to historical outcomes with azacitidine monotherapy,^22,40^ as well as at the time of progression from DNMTI monotherapy, where combination therapy may have encouraging activity.^38,41^

**Figure 1.**
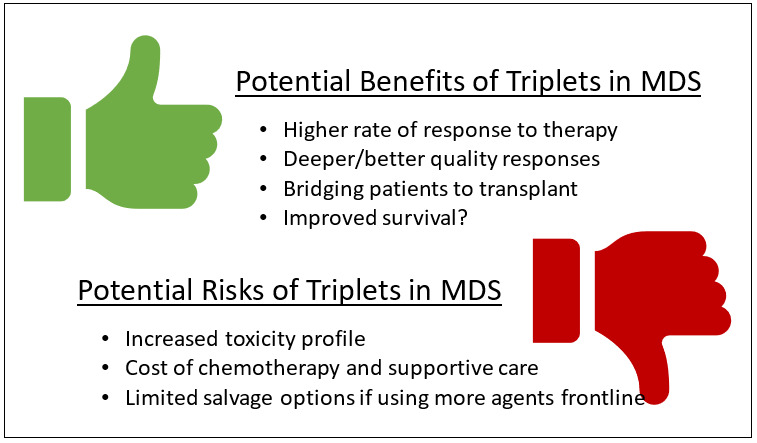
Potential Risks and Benefits of “Triplet” Combinations in HR-MDS.

Using this example, a number of questions arise as to how to most effectively build upon this backbone. Azacitidine monotherapy is typically initiated for HR-MDS and then continued ongoing until evidence of disease progression, and as such most doublets have taken a similar design, adding a second agent to every cycle of therapy.^42^ Should a triplet be utilized in a similar fashion, perhaps getting a response but then staying on all agents until progression? When explored in AML, the three drug regimen of decitabine, FLT3i, and venetoclax in *FLT3* mutant AML may yield high response rates in the front line (11 of 12 patients with CR (9) or CRp (2)) while more responses with incomplete count recovery were seen in relapse.^43^ Myelosuppression is an overlapping toxicity of all three agents in this cohort, and approximately 40% of treatment cycles were delayed due to cytopenias. In MDS, where doublets are yet to be proven and where a patient’s marrow failure component may be more significant at baseline, would a triplet regimen be better as initial therapy, sequenced or parallel, or intermittently held to allow for therapeutic holidays viewed as maintenance periods? Or instead, is it better to delay initiation of intensive multidrug regimens in this chronic disease, “saving” them until later lines of therapy (Figure 1)?^41^ Development of trials in this space may look to other hematologic malignancies common among older adults; for instance, in multiple myeloma, therapeutic windows have long been divided into induction, consolidation, and maintenance phases, each with their own focus.^44^ While the induction phase focuses on higher overall and complete response rates, studies for multiple myeloma maintenance focus more on long term tolerability and delaying relapse^45,46^; as it is unlikely that one combination therapy satisfies all these aims, new trials in MDS may have more success by segmenting treatment periods.

## Outcomes/Efficacy and Trial Regulatory “Success” with Triplet Combinations

One consideration around developing triplet therapies in MDS pertains to trial design and selecting appropriate endpoints for any clinical study. This is particularly challenging in MDS given the heterogeneity of the underlying disease as well as the patient population.^47^ In addition, compared to other myeloid neoplasms like AML, MDS is characterized by a dysfunctional marrow microenvironment contributing to ineffective hematopoiesis and deep cytopenias. A number of efforts have sought to streamline and improve avenues for drug development in MDS, particularly in higher-risk MDS where disease has overlapping features with AML.^48^ It is also apparent that endpoints in MDS are heterogeneous but some have stronger objective data than others; a patient-level analysis suggested that patients who achieve a strict CR, PR or HI had prolonged overall survival compared to non-responders, with the best being in patients with CR.^49,50^ Other meaningful endpoints include the duration of a given response to therapy, as well as overall survival, although these are later endpoints for clinical study.^48^ There are additional efforts underway to identify novel responses that are clinically meaningful and may serve as surrogates for overall survival, such as CRh (complete remission with incomplete hematologic recovery) or other combination endpoints,^51^ but these remain exploratory at this time. The recently published International Working Group (IWG) 2023 response criteria for HR-MDS constitute a major step towards establishing clinically meaningful endpoints but require prospective validation.^52^

Understanding these endpoints may provide some insights into triplet trial design. For instance, CR+PR has been utilized as a regulatory endpoint for prior studies in higher-risk MDS, and the response rates in MDS that have been reported with combination therapies may lend themselves to trials which use “triplets” early in disease to get a response, and then “de-escalate” the combination partners once in remission to limit cumulative toxicity. Unlike AML, therapy in MDS has not historically been divided into “remission induction,” “consolidation,” or “maintenance” periods, although doing so may support more innovative drug design, particularly with compounds that have overlapping toxicities. It should be noted, however, that CR is a fairly strict criterion; early phase studies may be particularly prone to overestimating activity in small numbers of patients, and appropriately powered phase II studies or randomized settings are needed to validate differences in response rates.^23^

It is also important to consider how crossover – whether planned or unintentional – may impact treatment design, particularly given the expansion of therapeutic options for the treatment of AML.^53^ In the case of venetoclax triplets this may be particularly important to include in the design of a trial; as venetoclax and azacitidine has efficacy for the treatment of AML, studies using this doublet in some ways are moving therapy earlier in disease, since patients with MDS who have progression on azacitidine monotherapy to AML could be considered for a venetoclax combination. Indeed, trials of an azacitidine+venetoclax±“Drug X” triplet in MDS may therefore be largely testing whether azacitidine+venetoclax is better utilized in the frontline (during MDS diagnosis) or second line (once they have AML).^54^ Some therapies may lend themselves more to randomized comparisons based on intention to treat; for instance, immune-based therapies, such as the anti-CD47 therapy magrolimab or the anti-TIM3 therapy sabatolimab, may have later impacts based on predicted immunological mechanisms of disease control.^55^ Such mechanisms of action may lend these therapies more for assessing the duration of response, or long term responses at the “tail” of the survival curve.

Whatever responses are seen in combination studies, a subsequent challenge will be to effectively disseminate this regimen in clinical practice. Current “real-life” use data suggests that only half of patients with MDS complete 4-6 cycles of azacitidine monotherapy,^56–58^ with some data suggesting this relates to comorbidities in this population as well as the logistical burden of administering intravenous or subcutaneous chemotherapy for 5-7 days every 4 weeks. It is possible that new oral formulations of DNMTIs, such as oral decitabine cedazuridine,^59^ will limit this clinical burden, and may thus facilitate adoption and adherence to new combinations. Indeed, completely oral therapy options for patients with MDS may improve both treatment persistence and also facilitate combination studies, by limiting the number of days requiring in-clinic chemotherapy administration. Lastly, in AML there is increasing recognition of the value of growth factor support and therapy holidays in the azacitidine+venetoclax treatment paradigm, which may also inform improved algorithms for MDS patients to avoid excessive toxicity from therapy.

## Conclusions

The current standard therapeutics in higher-risk MDS remain inadequate; too few patients will achieve complete remission, any responses to therapy are too short, and survival in HR-MDS is worse than most other advanced malignancies. Ongoing efforts continue to explore new active agents in this disease are underway, including doublet and triplet chemotherapy combinations. There are certain settings where “AML-like” therapies are appropriate in MDS, particularly given our evolving understanding of the overlapping biology of these malignancies. That said, a great deal more experience is needed before triplet therapies can become a reality for routine MDS care.

### Authors Contribution per CRediT taxonomy

Conceptualization: Andrew M. Brunner (Equal), Uwe Platzbecker (Equal), Amy E. DeZern (Equal), Amer M. Zeidan (Equal). Methodology: Andrew M. Brunner (Equal), Uwe Platzbecker (Equal), Amy E. DeZern (Equal), Amer M. Zeidan (Equal). Formal Analysis: Andrew M. Brunner (Equal), Uwe Platzbecker (Equal), Amy E. DeZern (Equal), Amer M. Zeidan (Equal). Investigation: Andrew M. Brunner (Equal), Uwe Platzbecker (Equal), Amy E. DeZern (Equal), Amer M. Zeidan (Equal). Writing – original draft: Andrew M. Brunner (Lead). Writing – review & editing: Andrew M. Brunner (Equal), Uwe Platzbecker (Equal), Amy E. DeZern (Equal), Amer M. Zeidan (Equal).

### Conflict of Interest

**Brunner:** Grant support: Edward P Evans Foundation. *Taiho:* Consultancy; *Takeda:* Consultancy, Research Funding; *Acceleron:* Honoraria; *Novartis:* Consultancy, Research Funding; *Keros Therapeutics:* Consultancy; *Janssen:* Research Funding; *GSK:* Research Funding; *Celgene/BMS:* Consultancy, Research Funding; *AstraZeneca:* Research Funding; *Agios:* Honoraria; *Aprea:* Research Funding.

**Platzbecker:***Janssen:* Honoraria; *Jazz:* Honoraria; *Silence Therapeutics:* Honoraria; *Takeda:* Honoraria; *Novartis:* Honoraria; *Abbvie:* Honoraria; *BMS/Celgene:* Honoraria; *Geron:* Honoraria.

**DeZern:***Gilead:* Consultancy, Honoraria; *GERON:* Other: DSMB; *Novartis:* Consultancy, Honoraria; *CTI BioPharma:* Consultancy, Honoraria; *Bristol Myers Squibb:* Consultancy, Honoraria; *Syntrix Pharmaceuticals:* Research Funding.

**Zeidan:** *BeyondSpring:* Consultancy; *Janssen:* Consultancy; *Boehringer Ingelheim:* Consultancy, Research Funding; *BioCryst:* Other: Clinical Trial Committees; *AstraZeneca:* Consultancy; *Pfizer:* Other: Travel support, Research Funding; *Kura:* Consultancy, Other: Clinical Trial Committees; *Incyte:* Consultancy, Research Funding; *Ionis:* Consultancy; *Daiichi Sankyo:* Consultancy; *Epizyme:* Consultancy; *Novartis:* Consultancy, Other: Clinical Trial Committees, Travel support, Research Funding; *Loxo Oncology:* Consultancy, Other: Clinical Trial Committees; *Genentech:* Consultancy; *Geron:* Other: Clinical Trial Committees; *Cardiff Oncology:* Consultancy, Other: Travel support, Research Funding; *BMS:* Consultancy, Other: Clinical Trial Committees, Research Funding; *Gilead:* Consultancy, Other: Clinical Trial Committees; *Aprea:* Consultancy, Research Funding; *Astellas:* Consultancy; *Astex:* Research Funding; *Jazz:* Consultancy; *Jasper:* Consultancy; *Amgen:* Consultancy, Research Funding; *Agios:* Consultancy; *ADC Therapeutics:* Research Funding; *Acceleron:* Consultancy, Research Funding; *AbbVie:* Consultancy, Other: Clinical Trial Committees, Research Funding.
